# Antimicrobial Resistance and Biofilm-Forming Ability in ESBL-Producing and Non-ESBL-Producing *Escherichia coli* and *Klebsiella pneumoniae* Isolated from Canine Urinary Samples from Italy

**DOI:** 10.3390/antibiotics14010031

**Published:** 2025-01-03

**Authors:** Alessia Facchin, Joel Filipe, Irene Mauri, Filippo Tagliasacchi, Guido Grilli, Tiziana Vitiello, Gabriele Ratti, Laura Musa, Martina Penati, Paola Scarpa, Stefania Lauzi

**Affiliations:** Department of Veterinary Medicine and Animal Sciences, University of Milan, Via dell’Università 6, 26900 Lodi, Italy; alessia.facchin@unimi.it (A.F.); guido.grilli@unimi.it (G.G.); laura.musa@unimi.it (L.M.); paola.scarpa@unimi.it (P.S.)

**Keywords:** dog, uropathogen, UTI, *E. coli*, *K. pneumoniae*

## Abstract

**Background:** In dogs, bacterial urinary tract infections are a frequent cause of antimicrobial prescription, increasing the risk of selecting antibiotic-resistant bacteria. This study analyzed resistance patterns, the presence of extended-spectrum β-lactamases (ESBLs) and biofilm-forming capacity in *E. coli* and *K. pneumoniae* previously isolated from urine samples collected from 133 selected dogs admitted to the Veterinary Teaching Hospital of Milan, Italy, in 2021 and 2023. **Methods**: The *E. coli* and *K. pneumoniae* isolates were bacteriologically and genetically analyzed. **Results**: Overall, 53/133 (39.8%) samples had a positive microbiological culture. Thirty-four *E. coli*/*K. pneumoniae* isolates were detected, accounting for 26.5% of the examined samples. The 34 isolates included 28 *E. coli* and 6 *K. pneumoniae*. Four (11.8%) were ESBL-producing bacteria, all supported by *bla*_CTX-M_ gene belonging to group 1. The *K. pneumoniae* isolates were significantly associated with ESBL production (*p* < 0.05). MIC analysis showed 11 (32.4%) multidrug-resistant isolates. Biofilm-forming capacity was observed in 23 (67.6%) isolates, regardless of bacterial species, including 20 weakly and 3 moderately adherent bacteria. All moderate biofilm producers were *K. pneumoniae*. Multidrug resistance (MDR) was significantly more present in strains with moderate biofilm-forming ability compared to strains with weak ability to form biofilm (*p* < 0.05). *E. coli* was confirmed as the most commonly identified urinary isolate in dogs. **Conclusions**: The high presence of ESBL producers and MDR in *K. pneumoniae* suggests mandatory in vitro susceptibility testing in the presence of this bacterium in dogs with UTI. The association of moderate biofilm production with MDR highlights the need for monitoring and surveillance of bacterial prevalence and resistance patterns of urinary isolates in dogs.

## 1. Introduction

Bacterial urinary tract infections (UTIs) are common in dogs, with 14% of the animals developing UTIs at some point in their lifetime [1,2]. The recommended diagnostic method for UTIs combines clinical signs, urinalysis—including dipstick testing, specific gravity measurement, and sediment cytology—and bacterial culture results obtained from urine samples collected using cystocentesis [3]. *Escherichia coli* (*E. coli*) is the most frequently detected pathogen, with a prevalence ranging from 35% to 64% [4,5]. Other common bacteria isolated from canine UTIs are *Staphylococcus* spp., *Enterococcus* spp., *Proteus mirabilis*, *Pseudomonas aeruginosa*, *Streptococcus* spp., and *Klebsiella* spp. [6].

The majority of UTIs are classified as sporadic bacterial cystitis, defined as a clinically apparent bladder infection occurring less than three times within a 12-month period that resolves with appropriate antimicrobial therapy [3,4]. Recurrent UTIs have been reported in dogs experiencing three or more episodes of clinical cystitis within the preceding 12 months, whereas animals with bacteriuria in the absence of clinical signs have been classified as having subclinical bacteriuria [3]. In 2019, the International Society for Companion Animal Infectious Diseases (ISCAID) released revised guidelines for the diagnosis and treatment of bacterial UTIs in pets [3,7].

UTIs are a common reason for antimicrobial prescription, and selection of antimicrobials to treat UTIs in dogs should be based on results of in vitro susceptibility testing [3]. The use of antimicrobials can drive the selection of resistant bacterial strains [8]. Infections caused by antimicrobial-resistant bacteria become harder to treat, and the risk of the spread of resistant bacteria increases. Among resistant bacteria, extended-spectrum β-lactamase (ESBL)-producing *E. coli* and *Klebsiella pneumoniae* have increasingly been reported in UTI [9]. Moreover, ESBL- and AmpC-producing *E. coli* and *Klebsiella* spp. are among important multidrug-resistant (MDR) bacteria [10]. Furthermore, biofilm formation plays a role in the development of complicated and recurrent UTI and may also act as a significant barrier to effective treatment, resulting in antimicrobial resistance (AMR) [11].

According to the World Health Organization (WHO), AMR is a growing global threat [12]. More recently, the emergence of carbapenemase (CP)-producing bacteria has been reported as a serious public health problem, and the presence of CP-producing *E. coli* has been documented in companion animals [13]. Additionally, given the close relationship between humans and pets, the potential for the transmission of antimicrobial-resistant bacteria from companion animals to humans, and vice versa, has been reported as a public health concern [6,14]. To obtain information useful for global plans aimed at fighting AMR, surveillance and rapid identification of AMR infections in pets have been highlighted within the framework of the One Health approach, which recognizes that the health of people is closely connected to the health of animals and the shared environment [14,15]. Bacterial prevalence and antimicrobial resistance patterns vary between medical facilities and across geographical regions [4], and data from Italy are limited.

The objectives of this study were to (i) retrospectively assess the presence of canine UTIs caused by *E. coli* and *K. pneumoniae*, (ii) identify the presence of ESBL-/AmpC/carbapenemase-producing *E. coli* and *K. pneumoniae* and evaluate their genotypic and phenotypic characteristics, and (iii) evaluate the antibiotic resistance profiles and assess biofilm formation in *E. coli* and *K. pneumoniae* isolates.

## 2. Results

### 2.1. Animal and Sample Collection

A total of 133 dogs’ urine samples were retrospectively selected from a collection of canine urine samples submitted to the Veterinary Teaching Hospital (VTH) of Lodi, University of Milan, following routine bacteriological analysis. Specifically, 56 (42.1%) samples were collected in 2021 and 77 (57.9%) in 2023. The dogs belonged to 42 different pure breeds, with the majority of breeds represented by one or two dogs. The pure breeds accounting for ≥4.5% (No. 6) of the dogs were Dachshund, German Shepherd, Golden Retriever, and Labrador Retriever. Data regarding the population characteristics of the dogs analyzed are summarized in Table 1.

### 2.2. Escherichia coli and Klebsiella pneumoniae Isolates

Out of the selected urine samples, 53 (39.8%) had a positive microbiological culture, defined as the isolation of at least one specific urinary bacterial pathogen [16]. In particular, 22/56 (39.3%) and 31/77 (40.3%) urine samples from the dogs were microbiologically positive in 2021 and 2023, respectively.

For the purpose of this study, only *E. coli* and *K. pneumoniae* isolates were considered, comprising 34 (25.6%, 95% CI: 18.2–33%) isolates. *E. coli* was detected in 28/133 (21.1%, 95% CI: 14.1–28%) and *K. pneumoniae* in 6 (4.5%, 95% CI: 1–8%) samples. More precisely, the 11 isolates collected in 2021 included 9 (16.1%) *E. coli* and 2 (3.6%) *K. pneumoniae*, while the 23 isolates collected in 2023 included 19 (24.7%) *E. coli* and 4 (5.2%) *K. pneumoniae*.

The characteristics of the positive dogs are reported in Table 1.

Most (90.9%) of the positive dogs with available clinical data had at least one of the clinical signs consistent with UTI, such as dysuria, pollakiuria, increased urgency, pyuria, and/or hematuria [3].

The results of the differences between the proportions of dogs with UTI caused by *E. coli* or *K. pneumoniae* and the characteristics of the dogs are reported in Table 1. The significant association of breeds with the presence of *E. coli*/*K. pneumoniae* reported in Table 1 was further confirmed by additional statistical analysis showing that Golden Retrievers were significantly more associated with *E. coli*/*K. pneumoniae* positivity compared to dogs belonging to other breeds or mixed breeds (*p* = 0.01), whereas other pure breeds were not significantly associated with higher positivity. Overall age groups were not significantly associated with UTIs caused by *E. coli* or *K. pneumoniae* (Table 1), and further statistical analysis showed a not significantly higher positivity in geriatric dogs compared to younger dogs (*p* = 0.0743).

### 2.3. Phenotypic and Genetic Characterization of ESBL-Producing E. coli and K. pneumoniae

Of the 34 strains screened, 4 (11.8%) tested positive for ESBL/AmpC production. Phenotypic characterization revealed that all isolates were positive exclusively for the ESBL phenotype, with no AmpC or carbapenemase production observed.

**Figure 1:**
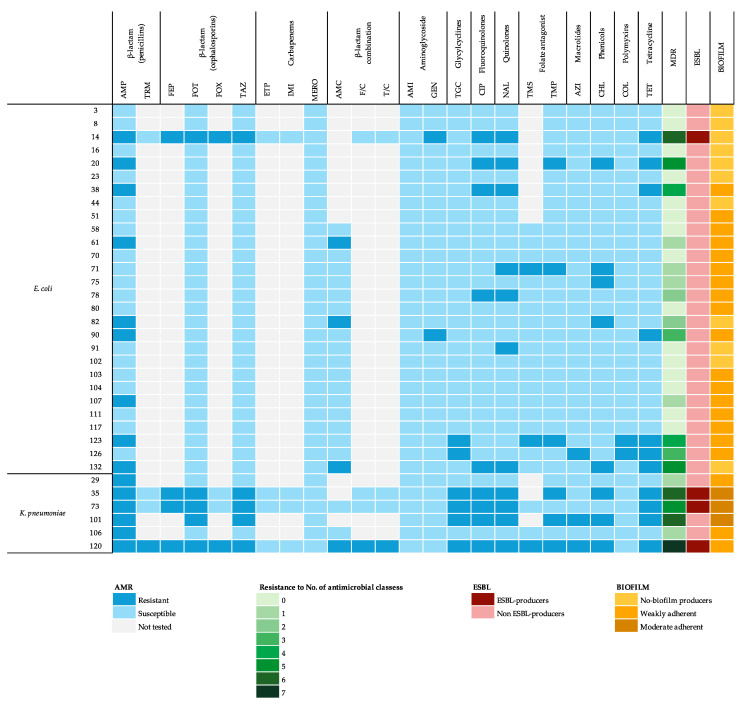
Heatmap illustrating the antimicrobial resistance profile of 28 *E. coli* and 6 *K. pneumoniae* strains. Rows represent individual bacterial isolates, while columns correspond to different antimicrobial agents categorized by class. The rightmost columns summarize MDR, ESBL production, and biofilm formation capacity. AMP: ampicillin; TRM: temocillin; FEP: cefepime; FOT: cefotaxime; FOX: cefoxitin; TAZ: ceftazidime; ETP: ertapenem; IMI: imipenem; MERO: meropenem; AMC: amoxiycillin/clavulanic acid; F/C: cefotaxime/clavulanic acid; T/C: ceftazidime/clavulanic acid; AMI: amikacin; GEN: gentamicin; TGC: tigecycline; CIP: ciprofloxacin; NAL: nalidixic acid; TMS: trimethoprim–sulfonamide; TMP: trimethoprim; AZI: Azithromycin; CHL: chloramphenicol; COL: colistin; TET: tetracycline.

The four ESBL-producing isolates consisted of one *E. coli* and three *K. pneumoniae* (Figure 1). All four ESBL-producing isolates were carriers of the *bla*_CTX-M_ gene belonging to group 1. All three *K. pneumoniae* isolates harbored *bla*_SHV_ and *bla*_TEM_ genes. Overall, the ESBL-producing *E. coli* and *K. pneumoniae* isolates were identified in 3% (95% CI: 0.1–5.9%) of the 133 urine samples. ESBL-producing bacteria were detected in 2/56 (3.6%) dogs in 2021 and 2/77 (2.6%) dogs in 2023.

ESBL-producing *E. coli* and *K. pneumoniae* were detected in 1/133 (0.75%, 95% CI: 0–2.2%) and 3/133 samples (2.3%, 95% CI: 0–4.8%), respectively. Table 1 shows the characteristics of dogs harboring ESBL-producing bacteria and the results of the differences between the proportions of dogs with and without ESBL-producing bacteria. Table 2 shows the characteristics of ESBL-producing bacteria and the results of the differences between the proportions between ESBL-producing bacteria and the characteristics of isolates.

### 2.4. Antimicrobial Minimum Inhibitory Concentration (MIC) and Disk Diffusion Testing

The resistance profiles of *E. coli* and *K. pneumoniae* isolates based on MIC and the disk diffusion test are presented in Figure 1 and Table 3. The distribution of MIC is reported in Table S1 for *E. coli* and Table S2 for *K. pneumoniae*.

Disk diffusion susceptibility testing results were available for 22 isolates, including 19/27 non-ESBL-producing *E. coli*, 1/3 non-ESBL-producing *K. pneumoniae*, and 2/3 ESBL-producing *K. pneumoniae* isolates. One out of the two ESBL-producing *K. pneumoniae* with available data on the disk diffusion test showed resistance to both amoxicillin/clavulanic acid and trimethoprim–sulfonamide.

Based on the MIC results, 11/34 (32.4%) MDR isolates were detected in total. Non-ESBL-producing *E. coli* and *K. pneumoniae* showed the presence of MDR isolates in six (22.2%) and one (33.3%) of the isolates, respectively. ESBL-producing bacteria were all MDR (Figure 1). Resistance to a number of antimicrobial classes is reported in Figure 1.

Overall, MDR isolates were detected in 11/133 (8.3%; 95% CI: 3.6–13%) of the samples analyzed.

Table 2 summarizes the results of MDR isolates according to the characteristics of the strains analyzed and the results of the difference in proportions between MDR bacteria and the characteristics of the isolates.

Additional statistical analysis on the association of MDR with the type of biofilm production showed that MDR was significantly more present in strains with moderate biofilm-forming ability compared to strains with weak ability to form biofilm (*p* = 0.03), whereas a not significant association was found when comparing these strains with the ones with no ability to form biofilm (*p* = 0.05).

### 2.5. Biofilm Formation

Out of the 34 bacterial strains, 23 (67.6%) isolates demonstrated biofilm-forming capability. In total, 20/34 (58.8%) strains exhibited weak adhesion, and 3 (8.8%) isolates showed moderate adhesion. The three strains with moderate biofilm-forming ability were collected from animals with recurrent infection. Table 1 and Table 2 summarize the results of biofilm production in the samples analyzed and in the isolates and the results of the difference in proportions between biofilm-producing bacteria and characteristics of the dogs and isolates. Biofilm-forming profiles are reported in Figure 1.

## 3. Discussion

In dogs, UTIs are a common reason for antimicrobial prescription posing the risk of selecting bacteria resistant to antibiotics. AMR is increasingly recognized as one of the most urgent public health threats globally, and the rising prevalence of ESBL-producing *Enterobacterales* has been reported as a significant threat to animal and human health [9,17]. The ability to form biofilm in strains isolated from dogs with UTI may also be associated with the presence of AMR [18]. This study analyzed resistance patterns, the presence of ESBL-producing bacteria, and the biofilm-forming capacity of *E. coli* and *K. pneumoniae* isolated from 133 selected urinary samples from dogs admitted to the VTH of the University of Milan, Italy, in 2021 and 2023.

The 39.8% prevalence of overall positive canine urine culture is similar to previous studies reporting a prevalence between 38% and 65% in dogs [19,20,21]. The presence of 21.1% *E. coli* and 4.5% *K. pneumoniae* is in accordance with previous reports indicating *E. coli* as the most frequently detected uropathogen in dogs and *K. pneumoniae* as a less frequently detected uropathogen [4]. However, the 21.1% *E. coli* positivity is lower compared to other studies showing *E. coli* prevalence ranging from 35% to 64% [2,4,22]. Comparison of the prevalence rates is difficult due to variations in the populations studied, the hospital demographics, or geographical location [4]. Further epidemiological research comparing the prevalence detected in our study with different studies in Italy would aid in better describing the prevalence of canine urinary *E. coli* isolates.

The higher *E. coli*/*K. pneumoniae* positivity of Golden Retriever dogs compared to other purebred dogs or mixed breeds confirms previous reports [23,24]. However, our results did not show a higher infection rate in German Shepherd or Labrador Retriever dogs, and this may be due to the low number of animals belonging to these breeds in this study or to other confounding factors. The absence of significant association of geriatric dogs with *E. coli*/*K. pneumoniae* infection was not expected given that previous studies reported that older animas may be predisposed to UTIs because they often experience a higher incidence of underlying health conditions and may have altered pharmacokinetics, making it more challenging to achieve adequate drug concentrations at infection sites [21,23,24]. Although UTIs have been reported to be more common in older female dogs, our results did not show that female dogs were at a major risk of *E. coli*/*K. pneumoniae* infection [23]. The presence of higher positivity of *E. coli*/*K. pneumoniae* infection in dogs with recurrent bacteriuria was expected, as persistent and recurrent UTIs are common in dogs and may prove difficult to treat using conventional antimicrobial therapy [23,24,25].

ESBL-producing bacteria in urine samples collected from the dog population and in the 34 isolates analyzed in this study have been previously described, although a higher prevalence was expected. However, it should be noted that most studies showing that the majority of ESBL-producing bacteria have been detected from the urinary tract of dogs have focused on the analysis of overall bacteria isolated from dogs from different sites [9]. Moreover, studies showing up to 37% of ESBL-producing bacterial isolates in patients with bacteriuria have focused on humans [26].

The consistent detection of *bla*_CTX-M_ group 1 in our isolates confirms that this is the most frequently detected ESBL gene in isolates from pets, as previously observed in France [27]. However, the prevalence of various ESBL in bacteria from companion animals may vary geographically, with *bla*_CTX-M-15_ as the most common in the US [9,28,29], whereas *bla*_CTX-M-14_ was most frequently detected in New Zealand [30]. The concurrent presence of *bla*_TEM_ and *bla*_SHV_ genes in *K. pneumoniae* isolates has been previously reported [31]. However, in the absence of the genetic characterization of the TEM and SHV types detected, our study does not allow for determining their contribution to the ESBL phenotype observed. Sequencing of these genes is needed because not all TEM-type and SHV-type β-lactamases display the ESBL phenotype [32]. The significantly higher presence of ESBL in *K. pneumoniae* compared to *E. coli* has been previously reported in humans, showing ESBL-producing *K. pneumoniae* among major pathogens in the outbreak of nosocomial infection, with 40% of clinical strains of *K. pneumoniae* being ESBL [33,34].

The high presence of bacteria with biofilm-forming ability, regardless of bacteria species, confirms that biofilm formation characterizes bacteria causing UTIs, with 60–80% of uropathogens causing UTI capable of forming a biofilm [18,35]. Biofilm formation enhances the ability to colonize the urinary tract while protecting the bacteria from the harsh bladder environment [18]. Biofilms may also promote persistence that can lead to chronic and recurrent UTIs [36], and our results showing that the three strains with moderate ability to form biofilm were collected from dogs with recurrent bacteriuria likely confirm this finding, although further investigations on a higher number of samples are needed.

The presence of MDR bacteria was expected despite the fact that a lower percentage of MDR was observed in this study compared to other investigations [37]. MDR highlights the risk of treatment failures and transmission of MDR bacteria to owners and the environment [38,39]. Antimicrobial treatment within the last month was confirmed as a risk factor associated with MDR, whereas hospitalization was not significantly associated with MDR [40,41]. Hospitalization and administration of antimicrobials are also known to be associated with ESBL-producing bacteria, but these risk factors were not found in this study, probably due to the low number of ESBL-producing bacteria detected [30,42]. The finding in our study of the association of recurrent bacteriuria with the presence of ESBL-producing bacteria compared to single infections has been previously reported, whereas the absence of significant association of recurrent bacteriuria with MDR was not expected and may be explained by the low number of isolates analyzed in this study. While uncomplicated UTI in dogs resolve within 3–10 days of treatment [3], recurrent infections are difficult to treat [23]. It cannot be excluded that antimicrobial therapy may have been administered before the present study, and treatment of recurrent UTI may have led to improper antimicrobial choices and the development of MDR bacteria [43].

The higher presence of MDR in *K. pneumoniae*, despite not being significant, along with resistance to a higher number of antimicrobial classes compared to *E. coli* was expected, given that *K. pneumoniae* multidrug-resistant types have been reported [44]. The significantly higher presence of MDR in ESBL-producing bacteria confirms previous findings [9].

The significant association between moderate biofilm-forming capacity and MDR observed in our study has been recently reported. Antimicrobial resistance phenotypes have been associated with biofilm formation in dogs [11,36]; however, recent studies have suggested that antimicrobial use may be selected for two populations: non-biofilm formers that maintain an arsenal of antimicrobial resistance genes to nullify treatment and a second that forms durable biofilms to avoid therapeutic insults [18]. The complex relationship between AMR and biofilms is not yet fully understood [45,46], and further investigations are needed.

In this study, *E. coli* and *K. pneumoniae* isolates were most likely to be resistant to ampicillin compared with other antimicrobials, as previously reported [6,47]. This result supports ISCAID indications not suggesting ampicillin as a first-line empirical therapy, despite its historical use as a primary treatment for UTIs in dogs [3,4]. Our results, which show resistance, even if at low levels, to ISCAID-recommended amoxicillin, amoxicillin/clavulanic acid—if amoxicillin without clavulanic acid is not readily available—and trimethoprim–sulfonamide as a first-line empirical antimicrobial for uncomplicated UTIs, question the efficacy of empirical treatment. Further epidemiological studies with a larger sample size are needed to evaluate the effectiveness and guide the empirical use of antimicrobials in Italy. Moreover, these results highlight the need for mandatory in vitro susceptibility testing, especially in the presence of *K. pneumoniae*, which are also likely ESBL producers with moderate biofilm-forming ability, and MDR.

The need for continuous monitoring for the emergence of resistance as a key component of AMR surveillance systems and evaluation of potential change in empirical treatment is warranted. A change in empirical treatment guidelines has been recommended when there is an increase in antimicrobial resistance in a non-biased population of 10% from baseline [48]. Considering that the change in antimicrobial resistance during the two years of our study was not investigated, prudent surveillance and monitoring are needed.

All isolates in this study were susceptible to amikacin, a finding of particular clinical relevance when considering local antimicrobial strategies for treating ESBL-producing Enterobacterales infections. However, given the potential nephrotoxicity associated with the use of aminoglycosides, their cautious use is recommended not only based on susceptibility test results but also considering the proper clinical assessment of the patient [3]. Particularly in cases of complicated UTIs where predisposing or determining factors cannot be eliminated, achieving a microbiological cure is often impossible. For this reason, it is prudent to reserve their use for critical situations involving the upper urinary tract or if there is a risk of sepsis [3].

The susceptibility of all isolates to carbapenems suggests their absence or low circulation of CP-producing bacteria among dogs, reducing potential health threats for humans. However, the presence of colistin resistance, considered a last-resort antibiotic for treatment of human infections, requires further investigations to confirm this finding, as colistin resistance has been reported in domestic animals, highlighting the need for intensive surveillance [49].

This study had some limitations. One limitation was that not all clinical data for all animals were available and colony-forming unit/mL was not considered, which could have helped in determining whether a true UTI was present compared to subclinical bacteriuria or contamination. Based on the isolates with clinical data available, it is likely that most of the isolates truly represented urinary tract infections and not bacteriuria. However, subclinical bacteriuria could not be ruled out in a portion of these patients for which clinical data were not available, and the possibility of clinically insignificant contamination in the free-catch urine sample collected from a patient with missing clinical data could not be completely ruled out. Another limitation was that only one sample was included from patients with multiple submissions in order to avoid biasing the population data with recurrent, resistant, or complicated UTIs. However, recurrent infections were observed in some dogs, and it cannot be ruled out that other samples were submitted in previous years given that complete information on dogs with recurrent UTIs was not included. On the other hand, evaluation of recurrent samples is needed because it could be of value to evaluate their resistance patterns, as these could differ [43]. Finally, identification of additional resistance genes and genetic characterization of isolates were not performed in this study, and future studies using whole genome sequencing (WGS) are warranted.

## 4. Materials and Methods

### 4.1. Animals and Bacterial Isolates

This study analyzed stored bacteria previously collected from canine urine samples. The samples were randomly selected from a collection of urine specimens submitted for routine microbiological analysis. These specimens were obtained from dogs admitted to the VTH of Lodi, University of Milan, in 2021 and 2023 Residual diagnostic samples, collected with the informed consent of the owners, were used for this study without any additional formal request for authorization, according to the decision of the Ethical Committee of the University of Milan (EC decision 29 October 2012, renewed with protocol no. 02-2016).

To focus on bacterial prevalence in single animals, only the initial positive urine culture from each patient was included. The canine urine samples were obtained via midstream voiding, catheterization, or cystocentesis and they were processed based on VTH microbiological routine analysis. For isolation, the samples were plated on selective agar plates, represented by blood agar and Brilliance™ UTI Agar (Thermofisher Scientific, Segrate, Milan, Italy), and colonies were selected and identified to the species level using the MBT Microflex LT/SH matrix-assisted laser desorption/ionization–time of flight mass spectrometry (MALDI-TOF MS) (Bruker Daltonik GmbH, Bremen, Germany), following previously described methods [50].

*E. coli* and *K. pneumoniae* colonies isolated from the selected samples and stored at −80 °C in Brain Heart Infusion (BHI) broth added with 15% glycerol were subjected to further analysis.

Information regarding sex, age, breed, weight, administration of antimicrobial therapy within the last 30 days, administered antimicrobial classes, hospitalization within the last 30 days, and presence of recurrent bacteriuria defined as ≥2 documented bacteriuria episodes in the last 6 months [51] was collected for each animal. The dogs were categorized into four age groups (Table S3): young (≤1 year), adult, senior, and geriatric age groups determined on the basis of weight classifications, as reported previously [52]. The breeds were divided into pure and mixed. The dogs were also categorized into the following four groups based on average adult weight: small breed (≤9 kg), medium breed (10 to 22 kg), large breed (23 to 41 kg), and giant breed (>41 kg) [52].

For the purpose of this study, a UTI was defined as the isolation of *E. coli* or *K. pneumoniae* from a urine sample.

### 4.2. Phenotypic Characterization of ESBL-/AmpC-/CP-Producing E. coli and K pneumoniae

*E. coli* and *K. pneumoniae* isolates were phenotypically characterized according to the guidelines published by EUCAST. Isolates were streaked on MacConkey agar supplemented with 1 mg/L cefotaxime [53] and incubated overnight at 37 °C. The suspected ESBL-producing isolates were subjected to phenotypic characterization in line with the European Committee on Antimicrobial Susceptibility Testing (EUCAST) guidelines [53]. Briefly, Mueller Hinton Agar (MH) was inoculated with standard inoculum (0.5 McFarland) of each isolate. ESBL production was confirmed using the combination disk test (CDT) with cefotaxime and ceftazidime disks alone or in combination with clavulanic acid (MAST Group Ltd., Bootle, UK). AmpC and CP production was tested using the AmpC detection set D69C and Carbaplus D73C (MAST Group Ltd., Bootle, UK), respectively. The Carbaplus D73C (MAST group Ltd., Bootle, UK) was used to identify KPC, metallo-β,-lactamase (MBL), or OXA-48 types. Plates were incubated at 37 °C for 18–24 h, based on the established protocols. After incubation, the inhibition zone surrounding each antimicrobial disk was measured and interpreted according to EUCAST guidelines [53] or the manufacturer’s instructions.

### 4.3. Genetic Characterization of ESBL-Producing E. coli and K. pneumoniae

According to the phenotypic test results, genetic characterization of *E. coli* and *K. pneumoniae* with ESBL-producing phenotypes was conducted. Bacterial DNA was extracted from all isolates using the boiling method (98 °C for 10 min). ESBL-encoding genes were detected using polymerase chain reaction (PCR) according to previously published protocols, with primers specific for the most common resistance genes: *bla*_CTX-M_, *bla*_TEM_, and *bla*_SHV_ [54]. The primer sequences used in this study are provided in Table 4.

For *bla*_CTX-M_-positive isolates, further PCR analysis was conducted to identify the *bla*_CTX-M_ group [55]. Each PCR amplification included both positive controls and blank controls (DNAse-free water) to ensure accuracy, and PCR products were visualized using gel electrophoresis on 1.5% agarose gels stained with ethidium bromide. The data obtained were used to assess the genetic profiles of the isolates and to understand the distribution of resistance genes within the collection.

### 4.4. Antimicrobial Minimum Inhibitory Concentration (MIC) and Disk Diffusion Susceptibility Testing

The minimum inhibitory concentrations (MICs) of antibiotics for *E. coli* and *K. pneumoniae* isolates were determined using the broth microdilution method using a commercial plate (Sensititre^TM^ EUVSEC 3, Thermo Scientific, Segrate, Milan, Italy), whereas ESBL-producing bacteria were also tested using an additional commercial plate (Sensititre^TM^ EUVSEC 2, ThermoScientfic, Segrate, Milan, Italy) in accordance with the guidelines established by the EUCAST, as described in Decision 2020/1729 EU [56]. Bacterial suspensions were adjusted to measure 0.5 McFarland concentration, and 10 μL of suspension was mixed in Mueller Hinton broth (MHB). The plates were inoculated with 50 μL of MHB containing bacteria and incubated at 37 °C for 18 h. The MIC values of each antibiotic were read using the Thermo Scientific Sensititre™ Manual Viewbox and interpreted according to the EUCAST human clinical breakpoint [57]. When EUCAST clinical breakpoints were not available, CLSI Vet01S [58] or EUCAST ECOFFs [59] were used.

Based on the MIC results, the isolates were classified as multidrug-resistant (MDR), defined as resistant to at least one agent in three or more antimicrobial categories [60].

Resistance to amoxicillin/clavulanic acid and trimethoprim–sulfonamide (TMS), recommended by ISCAID for empirical treatment for bacterial UTIs in dogs [3], was evaluated using, when available, the data from the routine microbiological analysis performed by the laboratory based on disk diffusion susceptibility testing, using CLSI breakpoints [58].

### 4.5. Biofilm Formation Assessment Using Microtiter Plate (MtP) Assay

Biofilm formation was assessed using a microtiter plate (MtP) assay, as previously described [61]. Briefly, after *E. coli* and *K. pneumoniae* cultures were grown in BHI broth at 37 °C for 24 h, they were diluted 1:100 in Tryptone Soya Broth (TSB) supplemented with 1% glucose and seeded in 96-well plates. After 24 h of incubation at 37 °C, planktonic bacteria were removed by gently washing the wells three times, and biofilm was fixed with 99% methanol and stained with Crystal Violet solution. The excess stain was removed by washing the wells, and the biofilm-bound crystal violet was solubilized with 70% ethanol. The negative control consisted of TSB + 1% glucose only. Each strain was analyzed in triplicate on the same plate, and three independent plates were used. The optical density (OD) at 570 nm for each well was measured using an Epoch ELISA reader (BioTek Instrumentals, Winooski, VT, USA). The optical density cut-off (ODc) was determined by calculating the average OD of the negative control plus the three standard deviations (SD) of the negative control. Biofilm formation by different strains was then analyzed and categorized as not, weakly, moderately, and strongly adherent based on the OD of the sample, as reported in Table S4 [61].

### 4.6. Sample Size Calculation and Statistical Analysis

In order to detect the presence of *E. coli* and *K. pneumoniae* in dogs, a sample size of 133 animals was calculated using WinEpi (http://www.winepi.net/uk/index.htm, accessed on 1 October 2023), considering a population size of 340 dogs admitted to the VTH in 2021 and 2023 for routine bacteriological analysis on urine samples and a minimum prevalence of 2%.

Pearson’s chi-square test or Fisher’s exact test, as appropriate, were used to evaluate the differences between the proportions of dogs with UTI caused by *E. coli*/*K. pneumoniae*, dogs with ESBL-producing *E. coli*/*K. pneumoniae*, and dogs with MDR *E. coli*/*K. pneumoniae* and sex, age groups, breed, breed size classification, administration of antimicrobial therapy, administered antimicrobial classes, hospitalization, recurrent bacteriuria, and year of sampling. Age was also further analyzed using Fisher’s exact test to evaluate the differences between the proportions of dogs with UTI caused by *E. coli*/*K. pneumoniae* and geriatric dogs compared to younger dogs. In the presence of a significant association of breed with dogs with UTI caused by *E. coli*/*K. pneumoniae*, Fisher’s exact test was also used to evaluate the proportions of positive dogs belonging to specific breeds compared to all other breeds. Fisher’s exact test was used to evaluate the differences between proportions of ESBL-producing bacteria, MDR bacteria, and biofilm-producing bacteria and the characteristics of the isolates. Fisher’s exact test was also used to evaluate proportions of MDR isolates according to the type of biofilm production. Statistical analysis was conducted using Epitools—Epidemiological Calculators, and a *p*-value ≤ 0.005 was considered statistically significant (https://epitools.ausvet.com.au/; accessed on 1 October 2024).

## 5. Conclusions

This study confirmed *E. coli* as the most commonly identified urinary isolate in dogs. The majority of isolates from the urine samples showed biofilm-forming ability, regardless of bacterial species, even if *K. pneumoniae* was more associated with moderate biofilm production. The results also highlight *K. pneumoniae* as a bacterium of concern, showing higher levels of ESBL production and MDR compared to *E. coli*. Continuous monitoring and surveillance of bacterial prevalence and resistance patterns in urinary isolates are essential for identifying future trends. Given the possibility of zoonotic transmission of antimicrobial-resistant bacteria, a One Health perspective concerning AMR spread is also needed in canine UTI cases.

## Figures and Tables

**Table 1:** Characteristics of dogs analyzed in this study and presence of *E. coli*/*K. pneumonia*, ESBL-producing bacteria, and MDR bacteria in the samples (No. = 133).

Population Characteristics		No.	No. (%) *E. coli*/ *K. pneumoniae*	Significance	No. (%) ESBL	Significance	No. (%) MDR	Significance
Sex	Male	53	12 (22.6)	0.53	1 (1.9)	0.873	4 (7.5)	1
	Female	80	22 (27.5)		3 (3.8)		7 (8.8)	
Breed ^a^	Dachshund	8	1 (12.5)	0.038 *	-	0.4846	-	0.074
	German Shepherd Dog	6	2 (33.3)		-		1 (16.7)	
	Golden Retriever	6	4 (66.7)		1 (16.7)		3 (50)	
	Labrador Retriever	7	2 (28.6)		-		-	
	Other pure breeds ^b^	69	11 (15.9)		2 (2.9)		3 (4.3)	
	Mixed breed	36	14 (38.9)		1 (2.8)		4 (11.1)	
Breed size classification	Small (≤9 kg)	43	8 (18.6)	0.3824	2 (4.7)	0.843	4 (10.3)	0.81
	Medium (10–22 kg)	39	11 (28.2)		1 (2.6)		3 (9.7)	
	Large (23–41 kg)	43	14 (32.6)		1 (2.3)		4 (12.1)	
	Giant (>41 kg)	8	1 (12.5)		-		-	
Age groups ^c^	Young (≤1 year)	15	3 (20)	0.331	-	0.469	1 (6.7)	0.45
	Adult (>1 to ≤4/6 years) ^d^	21	5 (23.8)		1 (4.8)		1 (4.8)	
	Senior (5/7 to 9/13 years) ^d^	59	13 (22)		3 (5.1)		6 (10.2)	
	Geriatric (≥10/14 years) ^d^	34	13 (38.2)		-		3 (8.8)	
Administration of antimicrobial therapy	Yes	35	13 (3.1)	0.07	2 (5.7)	0.169	6 (17.1)	0.037 *
	No	98	21 (21.4)		1 (1)		5 (5.1)	
Administered antimicrobial agents ^e^	Amoxicillin + clavulanic acid	19	1 (5.3)	nd	1 (5.3)	nd	1 (5.3)	nd
	Ampicillin	4	3 (75)		-		(33.3)	
	Ceftriaxone	1	-		-		-	
	Clindamycin	1	-		-		-	
	Doxycycline	1	-		-		-	
	Enrofloxacin	8	3 (37.5)		1 (12.5)		2 (25)	
	Marbofloxacin	5	4 (80)		2 (40)		3 (60)	
	Nitrofurantoin	1	1		1 (100)		1 (100)	
	Spiramycin + metronidazole	2	1		-		-	
Hospitalization	Yes	29	11 (37.9)	0.37	2 (6.9)	0.21	5 (17.2)	0.05
	No	104	23 (22.1)		2 (1.9)		6 (5.8)	
Recurrent infection	Yes	12	7 (58.3)	0.01 *	2 (16.7)	0.04 *	4 (33.3)	0.06
	No	121	27 (22.3)		2 (1.7)		7 (5.8)	
Type of sampling ^f^	Cystocentesis	117	32 (27.4)	0.61	4 (3.4)	0.905	9 (7.7)	0.493
	Catheterism	3	-		-		-	
	Voided sample	10	2 (20)		-		2 (20)	
Year of sampling	2021	56	11 (19.6)	0.18	2 (3.6%)	0.51	4 (56)	0.76
	2023	77	23 (29.9)		2 (2.6%)		7 (77)	

^a^ Breed was not known for one dog; ^b^ pure breeds accounting for <4.5% of dogs; ^c^ age was not known for four dogs; ^d^ according to age categories based on average adult weight (Table S3); ^e^ antimicrobial agent administered was not known for four dogs; ^f^ type of sampling was unknown for three dogs; -: absence of resistant isolates; nd: not determined; *: *p* < 0.05.

**Table 2:** ESBL production, MDR, and biofilm production according to bacterial characteristics.

Characteristics		No.	No. (%) ESBL	Significance	No. (%) MDR	Significance	No. (%) Biofilm-Producing	Significance
Bacterial species	*E. coli*	28	1 (3.6)	0.012 *	7 (25)	0.07	17 (60.7)	0.15
	*K. pneumoniae*	6	3 (50)		4 (66.7)		6 (100)	
ESBL production	Non-ESBL	30	nd		7 (23.3)	0.01 *	20 (66.7)	1
	ESBL-	4			4 (100)		3 (75)	
MDR isolates	Yes	11	4 (100)	0.01 *	nd		8 (72.7)	0.096
	No	23	-				15 (65.2)	
Biofilm- production	Non-biofilm producers	11	1 (9.1)	0.104	3 (27.3)	0.056	nd	
	Weakly adherent	20	1 (5)		5 (25)			
	Moderately adherent	3	2 (66.7)		3 (100)			

nd: not determined; *: *p* < 0.05.

**Table 3:** Number of *E. coli* and *K. pneumoniae* isolates resistant to antimicrobial agents based on MIC tests performed on 34 isolates, additional antimicrobial agents based on MIC tests performed on the four ESBL-producing bacteria, and disk diffusion testing results available for 22 isolates.

Antimicrobial Class	Antimicrobial Agent	Breakpoint	Total No. Resistant/ Total Analyzed (%)
β-lactam (penicillins)	AMP ^a^	8	15/34 (44)
	TRM ^a^	16	1/4 (25) ^e^
β-lactam (cephalosporins)	FEP ^a^	4	1/4 (25) ^e^
	FOT ^a^	2	5/34 (15)
	FOX ^a^	8	2/4 (50) ^e^
	TAZ ^a^	4	5/34 (15)
Carbapenems	ETP ^a^	0.5	0/4 (0) ^e^
	IMI ^a^	4	0/4 (0) ^e^
	MERO ^a^	8	0/34
β-lactam combination	AMC ^b^	17	4/22 (18)
	F/C ^c^	0.25	1/4 (25) ^e^
	T/C ^c^	1 (*E. coli*); 0.5 (*K. pneumoniae*)	1/4 (25) ^e^
Aminoglycoside	AMI ^a^	8	0/34 (0)
	GEN ^a^	2	2/34 (6)
Glycylcyclines	TGC ^a^	0.5	6/34 (18)
Fluoroquinolones	CIP ^a^	0.5	9/34 (27)
Quinolones	NAL ^c^	8	11/34 (32)
Folate antagonist	TMS ^b^	10	3/22 (14)
	TMP ^c^	2	6/34 (18)
Macrolides	AZI ^c^	16	3/34 (9)
Phenicols	CHL ^a^	16	8/34 (24)
Polymyxins	COL ^a^	2	2/34 (6)
Tetracycline	TET ^d^	16	11/34 (32)

^a^ MIC clinical breakpoint (μg/mL) reported by EUCAST was used; ^b^ data from in vitro susceptibility testing using zone diameter breakpoint (nearest wall mm) available for 19 non-ESBL-producing *E. coli*, 1 non-ESBL-producing *K. pneumoniae*, and 2 ESBL-producing *K. pneumoniae*; ^c^ MIC ECOFF (μg/mL) reported by EUCAST was used; ^d^ MIC clinical breakpoint (μg/mL) reported by Human CLSI was used; ^e^ only ESBL-producing bacteria were tested. AMP: ampicillin; TRM: temocillin; FEP: cefepime; FOT: cefotaxime; FOX: cefoxitin; TAZ: ceftazidime; ETP: ertapenem; IMI: imipenem; MERO: meropenem; AMC: amoxiycillin/clavulanic acid; F/C: cefotaxime/clavulanic acid; T/C: ceftazidime/clavulanic acid; AMI: amikacin; GEN: gentamicin; TGC: tigecycline; CIP: ciprofloxacin; NAL: nalidixic acid; TMS: trimethoprim–sulfonamide; TMP: trimethoprim; AZI: azithromycin; CHL: chloramphenicol; COL: colistin; TET: tetracycline.

**Table 4:** Primers used in this study.

Target	Primer	Sequence 5′-3′	Reference
CTX-M	*bla*_CTX-M_ F	ATGTGCAGYACCAGTAARGTKATGGC	[54]
	*bla*_CTX-M_ R	TGGGTRAARTARGTSACCAGAAYCAGCGG	
CTX-M-1 group	*bla*_CTX-M-1_ F	GGTTAAAAAATCACTGCGYC	[55]
	*bla*_CTX-M-1_ R	TYGGTGACGATTTTAGCCGC	
TEM	*bla*_TEM_ F	TCGCCGCATACACTATTCTCAGAATGA	[54]
	*bla*_TEM_ R	ACGCTCACCGGCTCCAGATTTAT	
SHV	*bla*_SHV_ F	ATGCGTTATATTCGCCTGTG	[54]
	*bla*_SHV_ R	TGCTTTGTTATTCGGGCCAA	

## Data Availability

The data that supported the findings of this study are available from the corresponding author upon reasonable request.

## Supplementary Materials

The following supporting information can be downloaded at https://www.mdpi.com/article/10.3390/antibiotics14010031/s1: Table S1: Distribution of MICs among *E. coli* isolates (No. = 28). White fields denote the range of dilutions tested for each antimicrobial agent, and gray fields denote the range of dilutions that were not tested for each antimicrobial agent. Vertical lines indicate the breakpoint used; Table S2: Distribution of MICs among *K. pneumoniae* isolates (No. = 6). White fields denote the range of dilutions tested for each antimicrobial agent, and gray fields denote the range of dilutions that were not tested for each antimicrobial agent. Vertical lines indicate the breakpoint used; Table S3: Classification of dogs into age groups based on weight categories; Table S4: Classification of biofilm formation abilities using the MtP method.
