# Supplementary material for: Antimicrobial Resistance and Biofilm-Forming Ability in ESBL-Producing and Non-ESBL-Producing Escherichia coli and Klebsiella pneumoniae Isolated from Canine Urinary Samples from Italy
Source: Antibiotics (Basel). 2025 Jan 3;14(1):31. doi: 10.3390/antibiotics14010031 (PMC11760867; doi:10.3390/antibiotics14010031)
Supplement: Supplementary file 1 [file antibiotics-14-00031-s001.zip › Table S2.pdf]

Table S2. Distribution of MICs among *K. pneumoniae* isolates (No. = 6). White fields denote range of dilutions tested for each antimicrobial agent, gray fields denote range of dilutions that were not tested for each antimicrobial agents. Vertical lines indicate the breakpoint used.

Antimicrobial class	Antimicrobial agent	No. resistant	0.0075	0.015	0.03	0.06	0.12	0.25	0.5	1	2	4	8	16	32	64	128	256	512	1024
B-lactam (penicillins)	AMP[a]	6/6													1	5				
	TRM[a]	1/3[g]												2	1					
B-lactam (cephalosporins)	FEP[a]	3/3[g]												1		2				
	FOT[a]	4/6						2					1				3			
	FOX[a]	1/3[g]										1	1		1					
	TAZ[a]	4/6						2						1	2		1			
Carbapenems	ETP[a]	0/3[g]				2			1											
	IMI[a]	0/3[g]						3												
	MERO[a]	0/6			4	1	1													
B-lactam combination agent	F/C[b]	1/3[g]					2			1										
	T/C[b]	1/3[g]						1		2										
Aminoglycoside	AMI[a]	0/6										5	1							
	GEN[a]	0/6							6											
Glycylcyclines	TGC[c]	4/6						1	1	2		2								
Fluoroquinolones	CIP[a]	4/6				2							1	3						
Quinolones	NAL[d]	4/6										2					4			
Folate antagonist	SMX[e]	ND/6												1	1					4
	TMP[b]	3/6							3						3					
Macrolides	AZI[d]	2/6												4	1	1				
Phenicols	CHL[a]	3/6											3		3					
Polymyxins	COL[a]	0/6								6										
Tetracycline	TET[f]	4/6									1	1				4				

[a] clinical breakpoint reported by EUCAST were used [57]; [b] ECOFF reported by EUCAST were used [59]; [c] clinical breakpoint of *E. coli* reported by EUCAST are used [57]; [d] ECOFF of *E. coli* reported by EUCAST were used [57]; [e] no breakpoint available; [f] clinical breakpoint reported by Human CLSI was used [58]; [g] Only ESBL-producing *K. pneumoniae* were tested; AMP: ampicillin; TRM: temocillin; FEP: cefepime; FOT: cefotaxime; FOX: cefoxitin; TAZ: ceftazidime; ETP: ertapenem; IMI: imipenem; MERO: meropenem; F/C: cefotaxime / clavulanic acid; T/C: ceftazidime / clavulanic acid; AMI: amikacin; GEN: gentamicin; TGC: tigecycline; CIP: ciprofloxacin; NAL: nalidixic acid; SMX: sulfamethoxazole; TMP: trimethoprim; AZI: Azithromycin; CHL: chloramphenicol; COL: colistin; TET: tetracycline; ND: not determined
