# Supplementary material for: Antimicrobial Resistance and Biofilm-Forming Ability in ESBL-Producing and Non-ESBL-Producing Escherichia coli and Klebsiella pneumoniae Isolated from Canine Urinary Samples from Italy
Source: Antibiotics (Basel). 2025 Jan 3;14(1):31. doi: 10.3390/antibiotics14010031 (PMC11760867; doi:10.3390/antibiotics14010031)
Supplement: Supplementary file 1 [file antibiotics-14-00031-s001.zip › Table S3.pdf]

Table S3. Classification of dogs into age groups based on weight categories.

Weight	Young	Adult*	Senior*	Geriatric*
≤9 kg (small breed)	≤ 1 year	>1 and ≤ 6 years	7 years – 13 years	≥ 14 years
10 - 22 kg (medium breed)	≤ 1 year	>1 and ≤ 6 years	7 years – 11 years	≥ 12 years
23 – 41 kg (large breed)	≤ 1 year	>1 and ≤ 5 years	6 year – 9 years	≥ 10 years
> 41 kg (giant breed)	≤ 1 year	>1 and ≤ 4 years	5 year – 9 years	≥ 10 years

* classification based on [52].
