# Supplementary material for: Antimicrobial Resistance and Biofilm-Forming Ability in ESBL-Producing and Non-ESBL-Producing Escherichia coli and Klebsiella pneumoniae Isolated from Canine Urinary Samples from Italy
Source: Antibiotics (Basel). 2025 Jan 3;14(1):31. doi: 10.3390/antibiotics14010031 (PMC11760867; doi:10.3390/antibiotics14010031)
Supplement: Supplementary file 1 [file antibiotics-14-00031-s001.zip › Table S4.pdf]

Table S4. Classification of dogs into age groups based on weight categories.

.Cut-off value calculation	Biofilm formation abilities*
OD ≤ ODc	Not adherent
ODc < sample OD ≤ 2 *x* ODc	Weakly adherent
2 *x* ODc < sample OD ≤ 4 *x* ODc	Moderately adherent
sample OD > 4 *x* ODc	Strongly adherent

* classification based on [61].
